# Resveratrol Ameliorates the Anxiety- and Depression-Like Behavior of Subclinical Hypothyroidism Rat: Possible Involvement of the HPT Axis, HPA Axis, and Wnt/β-Catenin Pathway

**DOI:** 10.3389/fendo.2016.00044

**Published:** 2016-05-24

**Authors:** Jin-Fang Ge, Ya-Yun Xu, Gan Qin, Jiang-Qun Cheng, Fei-Hu Chen

**Affiliations:** ^1^Anhui Key Laboratory of Bioactivity of Natural Products, School of Pharmacy, Anhui Medical University, Hefei, Anhui, China

**Keywords:** depression, hypothalamus–pituitary–adrenal axis, hypothalamic–pituitary–thyroid axis, resveratrol, subclinical hypothyroidism, Wnt/β-catenin pathway

## Abstract

Metabolic disease subclinical hypothyroidism (SCH) is closely associated with depression-like behavior both in human and animal studies, and our previous studies have identified the antidepressant effect of resveratrol (RES) in stressed rat model. The aim of this study was to investigate whether RES would manifest an antidepressant effect in SCH rat model and explore the possible mechanism. A SCH rat model was induced by hemi-thyroid electrocauterization, after which the model rats in the RES and LT4 groups received a daily intragastric injection of RES at the dose of 15 mg/kg or LT4 at the dose of 60 μg/kg for 16 days. The rats’ plasma concentrations of thyroid hormones were measured. Behavioral performance and hypothalamic–pituitary–adrenal (HPA) activity were evaluated. The protein expression levels of the Wnt/β-catenin in the hippocampus were detected by western blot. The results showed that RES treatment downregulated the elevated plasma thyroid-stimulating hormone concentration and the hypothalamic mRNA expression of thyrotropin-releasing hormone in the SCH rats. RES-treated rats showed increased rearing frequency and distance in the open-field test, increased sucrose preference in the sucrose preference test, and decreased immobility in the forced swimming test compared with SCH rats. The ratio of the adrenal gland weight to body weight, the plasma corticosterone levels, and the hypothalamic corticotrophin-releasing hormone mRNA expression were reduced in the RES-treated rats. Moreover, RES treatment upregulated the relative ratio of phosphorylated-GSK3β (p-GSK3β)/GSK3β and protein levels of p-GSK3β, cyclin D1, and c-myc, while downregulating the relative ratio of phosphorylated-β-catenin (p-β-catenin)/β-catenin and expression of GSK3β in the hippocampus. These findings suggest that RES exerts anxiolytic- and antidepressant-like effect in SCH rats by downregulating hyperactivity of the HPA axis and regulating both the HPT axis and the Wnt/β-catenin pathway.

## Introduction

Imbalances in thyroid hormone homeostasis are associated with both functional and structural brain alterations, resulting in neurobehavioral alterations, including depression (1, 2). Subclinical hypothyroidism (SCH) is defined as an elevated plasma thyroid-stimulating hormone (TSH) level associated with normal total or free thyroxine (fT4) and triiodothyronine (T3) levels. Although the hypometabolism symptoms, including fatigue, weakness, and cold intolerance, are dormant and non-specific in SCH patients, increasing evidence suggests that SCH is associated with neuropsychiatric disorders such as cognitive dysfunction (2) and depression (3, 4). Depression is observed more frequently among individuals with SCH than those with overt hypothyroidism (3), and SCH patients exhibit a twofold higher prevalence of depressive-like symptoms than healthy individuals (5). In our previous study, SCH induced depression-like behavior in rats accompanied by subtle hyperactivity of the hypothalamus–pituitary–adrenal (HPA) axis (6). Clinical studies have demonstrated that treatment with levothyroxine (LT4) improves mood and normalizes the elevated relative cerebral glucose metabolism in several brain areas of depression patients (7, 8). However, the correct dosage of LT4 remains elusive. Moreover, the unpredictable clinical effects of the currently available antidepressants, including poor efficacy and adverse reactions, make the development of new drugs to alleviate depression in SCH patients an urgent clinical need.

Resveratrol (*trans*-3,5,4′-trihydroxy-*trans*-stilbene, RES), a polyphenol component found mainly in grape and *Polygonum cuspidatum*, possesses multiple biological and pharmacological activities, including metabolism regulation (9) and antioxidant effects (10). Recently, our results (11), together with findings from other studies (12–15), have demonstrated that RES alleviates depression-like behavior in a rat model of chronic unpredicted mild stress (CUMS) through its antioxidant effects and by ameliorating hyperactivity of the HPA axis. In addition, the monoaminergic system and the molecular markers related to depression were also altered by RES treatment (16). However, it remains unknown whether RES can alleviate the depression-like symptoms in SCH, which is complicated with the balance of both the hypothalamic–pituitary–thyroid (HPT) axis and the HPA axis. Clinic studies showed that RES was well tolerated with the dose ranging from 200 mg (17) to 1000 mg (18) daily. According to the formula for dose translation based on body surface area (BSA), the corresponding dose in rats should range from 18 to 90 mg/kg. With regard to animal research, it has been reported that RES (20, 40, and 80 mg/kg) could significantly decreased the immobility time of mice in the despair tests (19). Consistently, results of our previous study also demonstrated that RES (15 mg/kg/day × 7 day) could significantly alleviate the depression-like behavior of CUMS rat (11). Thus, the dose of 15 mg/kg/day was chosen in this study.

Multiple approaches have been adopted to evaluate the antidepressant potential of compounds in animal models, including behavioral tests and biochemical/neurochemical assays. In rodents, spontaneous motor activity and anxiety are often analyzed in terms of exploratory behavior, especially during exposure to an open field (20). And the clinical symptoms/signs of depression such as anhedonia (incapability to perform rewarded behaviors) and helplessness are usually measured using the sucrose preference test (SPT) and the forced swimming test (FST), respectively (13). In this study, these behavior tasks were used, and the activity of the HPA axis was also detected.

Several studies in recent years have implicated the canonical Wnt signaling pathway in mood disorders such as bipolar disorder (21) and major depression (22). Activation of the canonical Wnt pathway leads to the inhibition of GSK-3β, allowing β-catenin to be stabilized in the cytosol and translocated to the nucleus, where it activates the transcription of target genes (23). Mutant mice with a heterozygous GSK-3β deletion showed decreased evidence of depression in the FST (23), and infusion of L803-mts, a selective GSK-3 inhibitor, also decreased immobility in the same test (24). Additionally, upregulated β-catenin has been used as a marker for antidepressive-like effects (24). Furthermore, thyroid hormone exerts a negative effect on the canonical Wnt signaling pathway, as demonstrated in a previous study (25). Thyroid hormone stimulates cell proliferation, represses the expression of key members of the Wnt signaling pathway, and suppresses β-catenin levels (25). Therefore, the Wnt/β-catenin pathway may participate in SCH-associated depression.

In this study, in order to explore the potential antidepressant-like effect of RES in the SCH rats and the possible mechanisms, behavior performance were evaluated using a series of behavioral tasks [open-field test (OFT), SPT, and FST]. Moreover, the activities of the HPA axis, the HPT axis, and the canonical Wnt pathway were assessed biochemically.

## Experimental Procedures

### Drugs

Resveratrol was purchased from Sigma Chemical Co. (St. Louis, MO, USA). LT4 was purchased from Berlin-Chemie AG (Berlin, Germany). Both drugs were dissolved in an aqueous solution of 0.5% sodium carboxymethyl cellulose to be a mixed suspension. Control and untreated model rats received a daily intragastric injection of 0.5% sodium carboxymethyl cellulose.

### Animals

Male, 2-month-old Sprague-Dawley rats were purchased from the Anhui Experimental Animal Center of China. They were housed three to four per cage (43 cm length × 31 cm width × 19 cm height) with access to food and water *ad libitum* and were maintained under a 12:12-h light/dark cycle. The light onset is at 8 o’clock. The ambient temperature was maintained at 21–22°C with 50–60% relative humidity. The rats were handled for 5 min daily over 7 days before drug administration. All experimental procedures in this study were approved by the Animal Care and Use Committee at the University of Science and Technology of China, which complies with the National Institute of Health Guide for the Care and Use of Laboratory Animals (NIH publication No. 85-23, revised 1985).

### Animal Model of SCH

The SCH rat model was established *via* hemi-electrocauterization, according to the procedures in our previous study (6). In brief, 33 rats underwent hemi-thyroid electrocauterization to establish the SCH model, and 8 sham rats underwent the same operation, but the thyroid tissues were exposed without electrocauterization. The SCH model was evaluated 2 weeks later, and the success rate of SCH modeling was 75.8% (25/33), according to the criterion that the plasma TSH concentration was higher than the 97.5 percentile of the sham group accompanied by a plasma fT4 level between the 2.5 and 97.5 percentile of the sham group. Consequently, 24 successful SCH rats were randomly divided into 3 groups with 8 rats in each group: an untreated model group, a RES treatment group (15 mg/kg/day + model) and an LT4 treatment group (60 μg/kg/day + model). The rats in the RES and LT4 groups received a daily intragastric injection of RES and LT4 at the corresponding dose for 16 days, respectively, and the rats in the sham and untreated model groups simultaneously received the same injection with 0.5% sodium carboxymethyl cellulose. To prevent hypocalcemia resulting from destruction of the parathyroid glands by electrocauterization, the rats were provided with 0.1% (w/v) calcium lactate in their drinking water after surgery.

### Behavioral Tests

**Figure 1:**
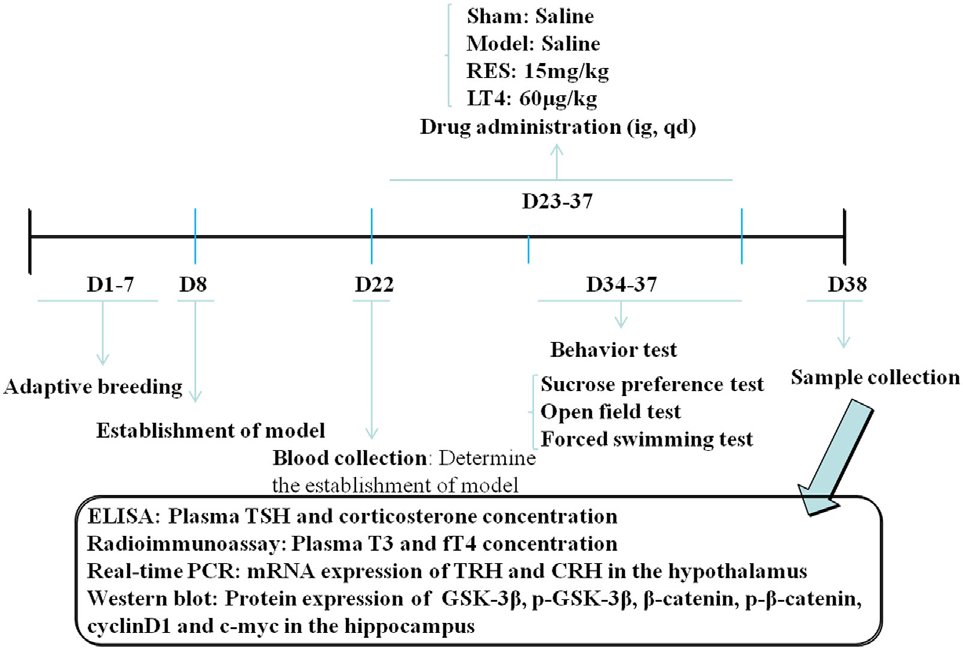
**Schedule of the experimental design**. RES, resveratrol; LT4, levothyroxine; T3, triiodothyronine; fT4, free thyroxine; CRH, corticotrophin-releasing hormone; TRH, thyrotropin-releasing hormone.

Behavioral tests were performed in a soundproof room with a neutral environment in the order listed in Figure 1. Briefly, SPT was carried out on day (D) 34, OFT on D 35, and FST on D 36 and D 37. All of the tests were carried out between 0900 and 1430 hours, with matching between the groups. The observers were blind to the treatment. The behavioral performance was monitored and recorded by a digital camera above the apparatus interfaced to a computer running the ANY-maze video imaging software (Stoelting Co., Wood Dale, IL, USA).

#### Sucrose Preference Test

After a 12-h period of food and water deprivation, the animals were individually housed in a cage (28 cm length × 17 cm width × 14.5 cm height) during the test and given two bottles (capacity: 250 ml) containing either water or a 2% sucrose solution. Six hours later, the volumes of water and sucrose consumed were measured. The percentage of the total liquid ingested was sucrose solution that was used as a measure of the sensitivity to reward.

#### Open-Field Test

The open-field apparatus consisted of a black square arena (100 cm × 100 cm), with a 30-cm-high wall. The floor was marked with a grid dividing it into 16 equally sized squares. During a 5-min observation period, the rat was placed at one corner of the apparatus facing the wall. After the 5-min test, rats were returned to their home cages, and the open field was cleaned with 75% ethyl alcohol and permitted to dry between tests. The total distance; average velocity; the distance, frequency, and duration in the center; and the frequencies of rearing, grooming, and defecation were recorded.

#### Forced Swimming Test

The behavioral cylinder was 60 cm high and 25 cm in diameter, maintained at 24–25°C, and filled with 30 cm of water, so that rats could not support themselves by touching the bottom with their paws. The FST paradigm includes two sections: an initial 15-min pretest followed by a 5-min test 24 h later. Rats were considered immobile when they did not make any active movements. Struggling was indicated when the rats made active movements with their forepaws in and out of the water along the side of the swim chamber. Swimming was indicated when the rats made active swimming or circular movements.

### Measurement of Plasma Concentrations of Thyroid Hormones and Corticosterone

Two weeks after the operation, blood samples (approximately 1 ml) were collected from the angular vein to test whether the SCH rat model was successfully established. Twenty-four hours after the last behavioral test, the rats were deeply anesthetized with chloral hydrate, and blood was taken from the abdominal aorta. Plasma concentrations of TSH and corticosterone were measured using ELISA kits (TSH: Cusabio Biotech. Co., Ltd., Wuhan, Hubei, China; corticosterone: Enzo Life Sciences, Inc., USA), according to the manufacturer’s instructions, and fT4 and T3 were measured with radioimmunoassay kits (North Institute of Biological Technology, Beijing, China), with the apparatus used in the assay that came from University of Science and Technology of China Zonkia (AnHui Ustc ZonKia Scientific Instruments Co., Ltd., Anhui China).

### The Ratio of the Adrenal Gland Weight to Body Weight

The adrenal glands of each side were removed and weighed immediately postmortem. The ratio of the average of both glands weight to body weight was measured.

### RNA Isolation and Real Time PCR

After blood collection, eight rats in each group were sacrificed by decapitation to collect the hypothalamus. The hypothalamus was rapidly dissected and frozen quickly in liquid nitrogen before storage at −80°C. Total RNA was extracted using the TRIzol (Invitrogen, Carlsbad, CA, USA) method. cDNA was synthesized using reverse transcriptase (Promega, WI, USA). Q-PCR was performed using the SYBR Green PCR Kit (Applied Biosystems, USA) and an ABI Prism 7000 Sequence Detector system in a 25-μl total reaction volume for 40 cycles (15 s at 95°C and 60 s at 62°C). The primers used in our study were as follows: rat β-actin 5′-TTGCTGACAGGATGCAGAA-3′ and 5′-ACCAATCCACACAGAGTACTT-3′; thyrotropin-releasing hormone (TRH) 5′-AGCTCAGCATCTTGGAAAGC-3′ and 5′-CCAGCAGCAACCAAGTC-3′; and corticotrophin-releasing hormone (CRH) 5′-CAGAACAACAGTGCGGGCTCA-3′ and 5′-AAGGCAGACAGGGCGACAGAG-3′. The relative amount of each target gene was calculated using the 2^−ΔΔCt^ method.

### Western Blot Assays

The hippocampus was homogenized in radioimmunoprecipitation assay (RIPA) buffer (50 mM Tris–HCl at pH 7.4, 0.1% SDS, 1% NP-40, 0.25% sodium deoxycholate, 150 mM NaCl, 1 mM EDTA, 1 mM EGTA, and 1 mM Na_3_VO_4_). Before homogenization, a protease inhibitor cocktail (Roche, IN, USA) and the phosphatase inhibitor PhosSTOP (Roche, IN, USA) were added. Protein quantitation was conducted using a Lowry Protein Assay Kit (Meiji Biotech Co., Ltd., Shanghai, China). The same quantity (approximately 50 μg) of protein from each animal was loaded and separated by 15% SDS-PAGE and then transferred onto a polyvinylidene difluoride membrane (Amersham Biosciences, UK). The membrane was blocked with 5% skim milk for 1 h, incubated with antibodies targeting GSK3β, p-GSK3β (Ser9), β-catenin, p-β-catenin (1:1000; Cell Signaling Technology, USA), cyclin D1, c-myc (1:1000; ImmunoWay, Newark, DE, USA), or β-actin (1:1000; Bioworld Technology, Inc., USA) at 4°C overnight, and then incubated with a horseradish peroxidase-conjugated secondary antibody (1:10,000) at 37°C for 1 h. The blots were developed with the Easy Enhanced Chemiluminescence Western Blot Kit (Pierce Biotechnology, Rockford, IL, USA). The protein bands were scanned and analyzed using Image J software (NIH).

### Statistical Analyses

All statistical analyses were performed using SPSS (Statistical Package for the Social Sciences) version 12.0.1 (SPSS Inc., Chicago, IL, USA). The data are expressed as the means ± SEM, and *P* < 0.05 was considered statistically significant. The distribution of the data was determined by the Kolmogorov–Smirnov test. Between-group effects on body weight, TSH, fT4, and T3 were analyzed by repeated measures ANOVA with group and days as the factors. Statistical analyses of the between-group effects of RES on behavioral performance, the ratio of the adrenal gland weight to body weight, plasma corticosterone, the hypothalamic mRNA expression of CRH and TRH, and the hippocampal protein expression of GSK3β, p-GSK3β (Ser9), β-catenin, p-β-catenin, cyclin D1, and c-myc were carried out using ANOVA followed by LSD *post hoc* tests. Correlation analysis was performed using a Pearson correlation test.

## Results

### RES Administration Decreased the Elevated Plasma TSH and the Hypothalamic TRH mRNA in SCH Rats

The repeated measures ANOVA revealed that both the treatment [*F*(5,140) = 11.975, *P* < 0.001] and time [*F*(3,28) = 76.925, *P* < 0.001] had a significant effect on the TSH levels, with no significant interaction [*F*(5,140) = 0.583, *P* = 0.614]. When it comes to the T3 levels, the results showed that neither the time [*F*(5,140) = 0.555, *P* = 0.461] nor the treatment [*F*(3,28) = 0.673, *P* = 0.574] had a significant effect, with no significant interaction [*F*(5,140) = 0.452, *P* = 0.718]. Moreover, repeated measures ANOVA revealed a significant interaction between the treatments and weeks in body weight [*F*(15,140) = 23.989, *P* < 0.001]. The individual factor treatment also had a significant effect [*F*(5,140) = 26.113, *P* < 0.001], but the factor time did not [*F*(3,28) = 2.509, *P* = 0.122].

**Figure 2:**
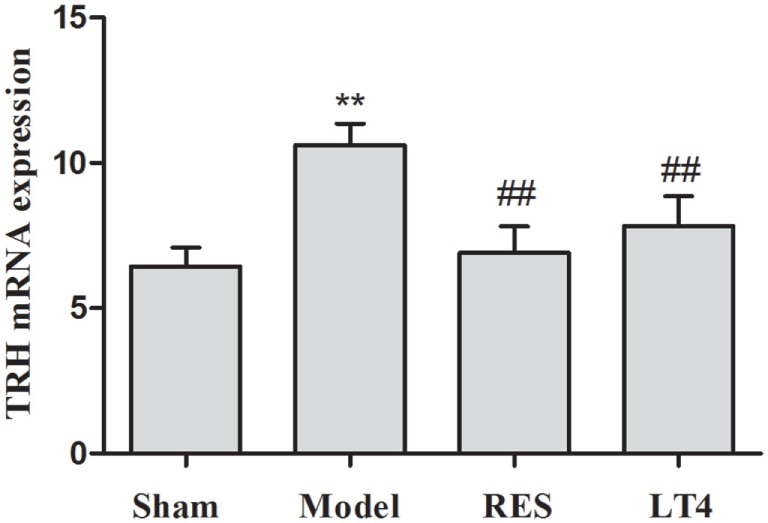
**Effect of RES on hypothalamic TRH mRNA expression in the SCH rats**. The mRNA expression of TRH in the hypothalamus is illustrated. The data are presented as the means ± SEM, with *n* = 8 for each group. *P < 0.05 and **P < 0.01 compared to the sham group. ^#^*P* < 0.05 and ^##^*P* < 0.01 compared to the untreated model group. RES, resveratrol; TRH, thyrotropin-releasing hormone; SCH, subclinical hypothyroidism.

As shown in Table 1, before the treatment, SCH rats in the untreated model, RES and LT4 groups showed elevated plasma TSH level [*F*(3,28) = 13.263, *P* < 0.01; LSD: sham vs. untreated model: *P* = 0.002, sham vs. RES: *P* < 0.001, sham vs. LT4: *P* < 0.001] with normal plasma fT4 [*F*(3,28) = 0.737, *P* = 0.539; LSD: sham vs. untreated model: *P* = 0.378, sham vs. RES: *P* = 0.689, sham vs. LT4: *P* = 0.876] and T3 [*F*(3,28) = 1.505, *P* = 0.235; LSD: sham vs. untreated model: *P* = 0.778, sham vs. RES: *P* = 0.701, sham vs. LT4: *P* = 0.423] concentrations compared with the sham ones. After 16 days of treatment, both RES and LT4 decreased the elevated TSH level of untreated model rats [*F*(3,28) = 11.269, *P* < 0.01; LSD: RES vs. untreated model: *P* = 0.028, LT4 vs. untreated model: *P* < 0.01]. No significant difference was found between sham rats and RES- or LT4-treated rats (LSD: RES vs. sham: *P* = 0.213, LT4 vs. untreated model: *P* = 0.711). Consistent with this result, the hypothalamic mRNA expression of TRH was inhibited by treatment with RES or LT4 [Figure 2, *F*(3,16) = 6.668, *P* < 0.001; LSD: RES vs. untreated model: *P* = 0.050, LT4 vs. untreated model: *P* = 0.01, RES vs. sham: *P* = 0.221, LT4 vs. untreated model: *P* = 0.611]. No significant difference was observed between the plasma T3 concentrations of the groups [Table 1, *F*(3,28) = 2.563, *P* = 0.458]. LT4, but not RES, increased the plasma fT4 concentrations compared to sham or untreated model rats [Table 1, *F*(3, 28) = 60.583, *P* < 0.01; LSD: RES vs. sham: *P* = 0.202; LT4 vs. sham: *P* < 0.001; RES vs. untreated model: *P* = 0.340, LT4 vs. untreated model: *P* < 0.001].

**Table 1:** **Concentrations of plasma total triiodothyronine (T3), free thyroxine (fT4), thyroid-stimulating hormone (TSH) in sham (*n* = 8), model (*n* = 8), RES (*n* = 8), and LT4 (*n* = 8) before and after treatment**.

Group	*n*	T3 (nmol/L)		fT4 (ρmol/L)		TSH (mIU/L)	
		Before	After	Before	After	Before	After
Sham	8	0.78 ± 0.03	0.72 ± 0.04	9.45 ± 0.34	5.78 ± 0.51	0.45 ± 0.06	0.46 ± 0.05
Model	8	0.75 ± 0.04	0.74 ± 0.05	9.44 ± 0.41	6.36 ± 0.40	2.28 ± 0.34**	1.31 ± 0.03**
RES	8	0.78 ± 0.05	0.66 ± 0.16	9.36 ± 0.49	6.84 ± 0.53	2.58 ± 0.29**	0.55 ± 0.09^#^
LT4	8	0.79 ± 0.02	0.81 ± 0.05	8.91 ± 0.25	15.67 ± 0.85**^,##^	2.68 ± 0.28**	0.09 ± 0.01^##^

*The data were presented as mean ± SEM*.

**P < 0.01 compared with sham group.

*^#^*P* < 0.05 and ^##^*P* < 0.01 compared with model group*.

*RES, resveratrol; LT4, levothyroxine*.

### RES Administration Did Not Reverse the Decreased Bodyweight in the SCH Rats

**Figure 3:**
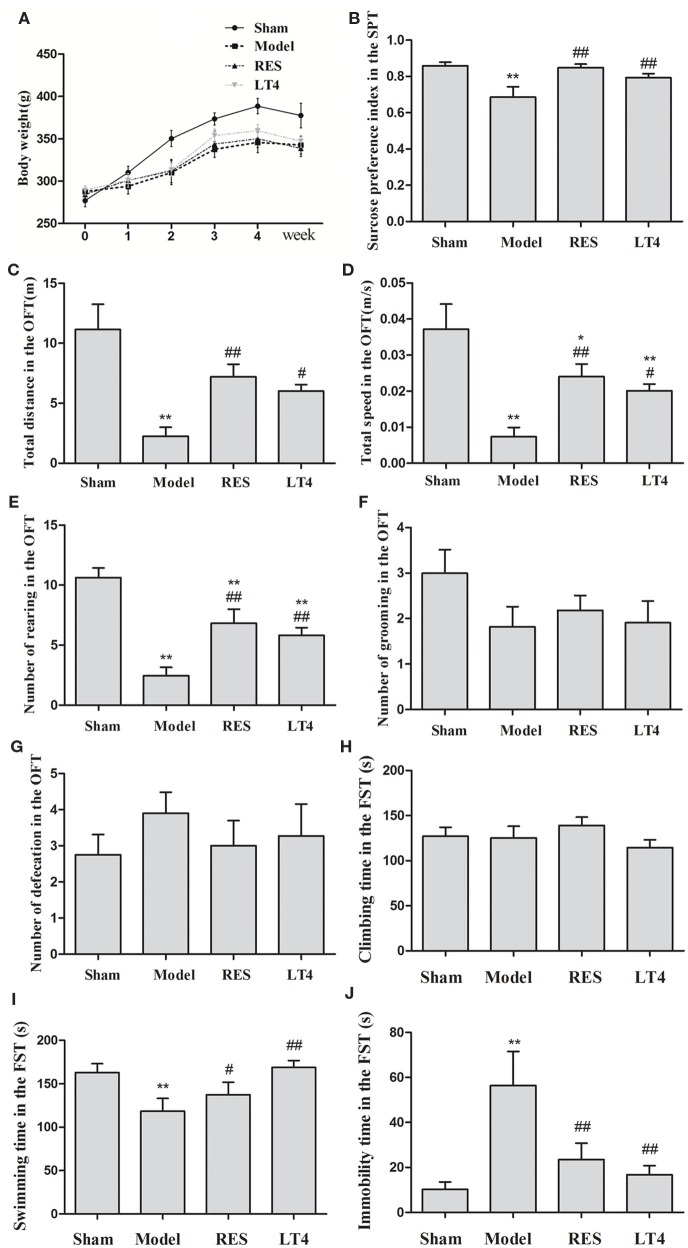
**Effect of RES on the body weight and behaviors of the SCH rats**. The body weight **(A)**, sucrose preference **(B)**, and performance in the OFT **(C–G)** and FST **(H–J)** were observed. The data are presented as the means ± SEM, with *n* = 8 for each group.^*^ *P* < 0.05 and **P < 0.01 compared to the sham group. ^#^*P* < 0.05 and ^##^*P* < 0.01 compared to the untreated model group. RES, resveratrol; SCH, subclinical hypothyroidism; OFT, open-field test; FST, forced swimming test.

Figure 3A shows the effect of RES on the body weight and behaviors of the SCH rats. Repeated measures ANOVA revealed a significant interaction between the treatments and weeks in body weight [*F*(15,140) = 3.707, *P* < 0.01]. The individual factor weeks also had a significant effect [*F*(5,140) = 165.550, *P* < 0.01], but the factor treatments did not [*F*(3,28) = 1.633, *P* = 0.198]. A significant difference in body weight was noticed in week 5 in the sham rats compared to the untreated model rats [*F*(3,28) = 3.179, *P* = 0.035; LSD: sham vs. untreated model: *P* = 0.027, RES vs. untreated model: *P* = 0.785, LT4 vs. untreated model: *P* = 0.745, RES vs. sham: *P* = 0.015, LT4 vs. untreated model: *P* = 0.052]. However, no change in body weight was observed during the experimental period in the untreated model rats compared to the RES- or LT4-treated rats, indicating that neither drug had an effect on the body weights of untreated model rats.

### RES Administration Alleviated the Anxiety- and Depressive-Like Behavior in the SCH Rats

The sucrose preference of the untreated model rats was remarkably lower than that of the sham group. Both the RES and LT4 groups showed an elevated sucrose preference index compared to the untreated model rats [Figure 3B, *F*(3,28) = 6.387, *P* = 0.001; LSD: RES vs. untreated model: *P* = 0.002, LT4 vs. untreated model: *P* = 0.035, RES vs. sham: *P* = 0.849, LT4 vs. untreated model: *P* = 0.224], indicating an anti-anhedonia effect of RES and LT4.

In the OFT (Figures 3C–G), the untreated model group traveled over a shorter total distance [*F*(3,28) = 9.983, *P* < 0.01; LSD: RES vs. untreated model: *P* = 0.002, LT4 vs. untreated model: *P* = 0.018, RES vs. sham: *P* = 0.022, LT4 vs. untreated model: *P* = 0.004] and at a lower velocity [*F*(3,28) = 9.987, *P* < 0.01; LSD: RES vs. untreated model: *P* = 0.002, LT4 vs. untreated model: *P* = 0.017, RES vs. sham: *P* = 0.023, LT4 vs. untreated model: *P* = 0.004] than the sham group, and these measures were ameliorated by the RES and LT4 treatments. These results indicate that both drug treatments improved the SCH rats’ locomotor activity. Moreover, a decreased number of rearing was observed in the untreated model rats, and this behavior was increased in the SCH rats treated with RES or LT4 [*F*(3,28) = 13.560, *P* < 0.01; LSD: RES vs. untreated model: *P* = 0.001, LT4 vs. untreated model: *P* = 0.008, RES vs. sham: *P* = 0.006, LT4 vs. untreated model: *P* = 0.001], indicating that both drug treatments improved exploratory behavior. As the total distance and frequency of rearing are also used as measures of anxiety (26), these data revealed that the drug treatments may have decreased the high anxiety levels in the untreated model rats. However, no differences in the number of grooming behaviors and defecations were observed between the SCH rats with and without RES treatment.

In the FST (Figures 3H–J), the untreated model rats spent a longer time immobile [*F*(3,28) = 4.792, *P* < 0.01; LSD: RES vs. untreated model: *P* = 0.014, LT4 vs. untreated model: *P* = 0.004, RES vs. sham: *P* = 0.351, LT4 vs. untreated model: *P* = 0.646] and less time swimming [*F*(3,28) = 3.557, *P* = 0.023; LSD: RES vs. untreated model: *P* = 0.014, LT4 vs. untreated model: *P* = 0.004, RES vs. sham: *P* = 0.351, LT4 vs. untreated model: *P* = 0.646], and these changes were reversed by RES or LT4 treatment, indicating that RES alleviated the despairing behavior in the SCH rats.

### RES Administration Decreased the Ratio of the Adrenal Gland Weight to Body Weight, Plasma Corticosterone, and Hypothalamic CRH mRNA in SCH Rats

**Figure 4:**
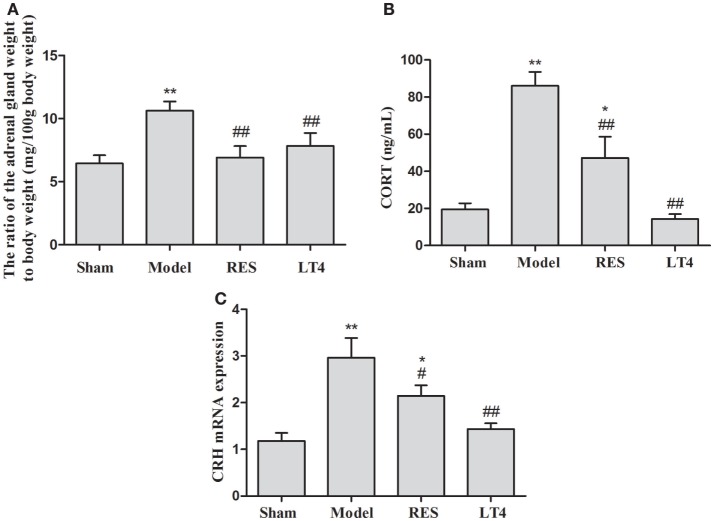
**Effects of RES on the ratio of the adrenal gland weight to body weight, the plasma corticosterone, and the expression of CRH mRNA in the hypothalamus in SCH rats**. The ratio of the adrenal gland weight to body weight **(A)**, the plasma corticosterone **(B)**, and the expression of CRH mRNA in the hypothalamus **(C)** are shown. The data are presented as the means ± SEM, with *n* = 8 for each group. *P < 0.05 and **P < 0.01 compared to the sham group. ^#^*P* < 0.05 and ^##^*P* < 0.01 compared to the untreated model group. RES = resveratrol, SCH = subclinical hypothyroidism, CRH = corticotrophin-releasing hormone.

Although the ratio of the adrenal gland weight to body weight was significantly increased in the untreated model rats (Figure 4A), both RES and LT4 significantly decreased the ratio compared to the sham group [*F*(3,28) = 22.777, *P* < 0.001; LSD: sham vs. untreated model: *P* < 0.001, RES vs. untreated model: *P* < 0.001, LT4 vs. untreated model: *P* < 0.001, RES vs. sham: *P* = 0.011, LT4 vs. untreated model: *P* = 0.604]. After the hemi-thyroid electrocauterization, plasma corticosterone in the untreated model rats increased significantly, while treatment with either RES or LT4 decreased the elevated corticosterone levels [Figure 4B, *F*(3,28) = 19.066, *P* < 0.01]. In line with this result, the elevated hypothalamic CRH mRNA expression noted in the untreated model rats was decreased by treatment with either RES or LT4 [Figure 4C, *F*(3,28) = 9.468, *P* < 0.01].

### RES Administration Decreased Activation of the Canonical Wnt Pathway in the Hippocampus of SCH Rats

**Figure 5:**
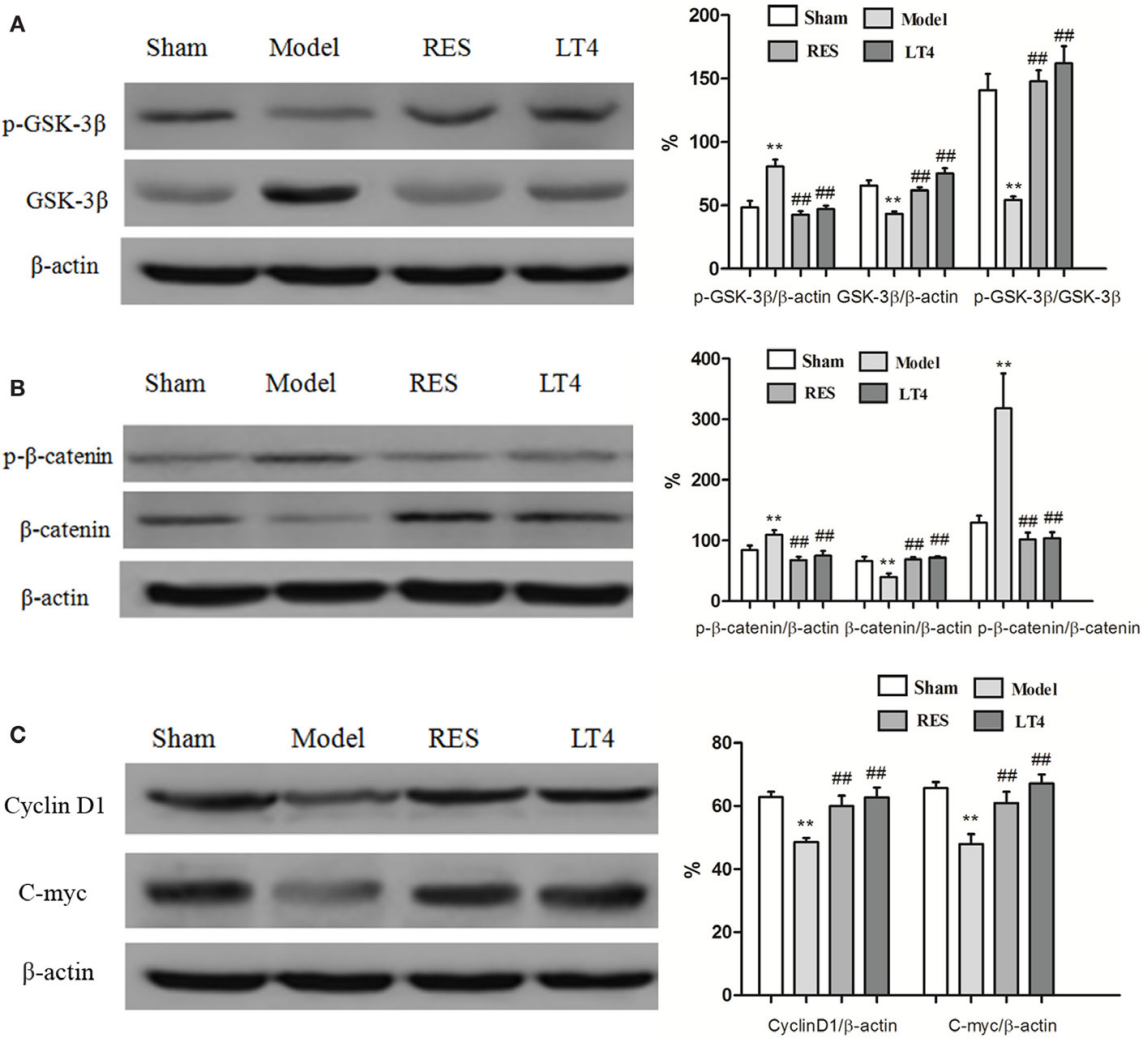
**Effects of RES on the activation of the canonical Wnt pathway in the hippocampi of SCH rats**. Representative blots and the quantitative analysis of GSK3β and p-GSK3β **(A)**, β-catenin and p-β-catenin **(B)**, cyclin D1 and c-myc **(C)** are presented. The data are presented as the means ± SEM, with *n* = 8 for each group. *P < 0.05 and **P < 0.01 compared to the sham group. ^#^*P* < 0.05 and ^##^*P* < 0.01 compared to the untreated model group. RES, resveratrol; SCH, subclinical hypothyroidism.

Figure 5 shows the protein expression levels of GSK-3β, p-GSK-3β (Ser9), β-catenin, p-β-catenin, cyclin D1, and c-myc in rat hippocampi. Compared with those of the sham group, a lower protein expression of p-GSK-3β [Figure 5A, *F*(3,28) = 23.969, *P* < 0.01] and relative ratio of p-GSK-3β/GSK-3β [*F*(3,28) = 21.902, *P* < 0.01] and a higher protein expression of GSK-3β [*F*(3,28) = 23.673, *P* < 0.01] were observed in the hippocampus of the untreated model rats, which was reversed by the RES or LT4 treatment.

As shown in Figure 5B, a higher protein expression of p-β-catenin [Figure 5B, *F*(3,28) = 11.322, *P* < 0.01] and relative ratio of p-β-catenin/β-catenin [Figure 5B, *F*(3,28) = 11.641, *P* < 0.01] were observed in the hippocampus of the untreated model rats. Conversely, the protein expression of β-catenin [Figure 5B, *F*(3,28) = 7.44, *P* = 0.002] was lower than that of the sham group. These changes were reversed by treatment with RES or LT4.

Consistently, hippocampal protein levels of cyclin D1 [Figure 5C, *F*(3,28) = 7.457, *P* < 0.01] and c-myc [*F*(3,28) = 8.922, *P* < 0.01] were lower in the untreated model rats than in the sham ones, but these changes were improved by treatment with RES or LT4. Altogether, these results indicate that the canonical Wnt pathway was activated in the hippocampus of the untreated model rats and that activation was ameliorated by the RES treatment.

Results of Pearson correlation analysis showed that the expressions of p-β-catenin/β-catenin were both negatively correlated to cyclin D1 (*r* = −0.566, *P* = 0.004) or c-myc (*r* = −0.565, *P* = 0.004).

## Discussion

In this study, we explored the antidepressant effects of RES in SCH rats. The results showed that RES treatment could alleviate anxiety- and depression-like behavior in SCH rats, as indicated by their increased rearing frequency and moving distance in the OFT, their elevated sucrose preference index, and their decreased immobility in the FST. Moreover, RES treatment improved the imbalance of HPA and HPT axes observed in the SCH rats. Furthermore, RES treatment downregulated activation of the canonical Wnt pathway in the hippocampus of SCH rats.

Subclinical hypothyroidism is a common thyroid dysfunction that occurs in 4–20% of the adult population. The risk of SCH progressing to overt hypothyroidism is approximately 7% (27), and both overt and SCH are associated with an increase in the number and severity of depressive-like symptoms (28,  29). LT4 is the routine clinical treatment for SCH. In this study, our results show that the imbalance of the HPT axis in SCH rats was improved by the treatment of LT4, as indicated by the decrease of both the plasma TSH and the hypothalamic TRH mRNA expression. However, the possibility of overtreatment is one of the adverse effects of LT4 treatment, and the risk ranges from 14 to 21% (30). Consistently, with a dose parallel to the routine dose used clinically, LT4-treated SCH rats showed significantly higher plasma fT4 concentration than the sham rats in our study.

The adverse side effects of LT4, including the hyperthyroxinemia, make the development of new therapeutic drugs to treat SCH necessary. Recently, a new concept of antidepressant mechanisms of action has been proposed based on the findings that antidepressants showed immediate antioxidant effects in the treatment of major depressive disorder (31). Increasing evidence from animal studies suggests that treatment with antioxidants can reduce oxidative stress and alleviate depressive-like behaviors (32). RES is a polyphenol antioxidant that has versatile biological and pharmacological activities, including neuroprotective effects (19, 33). Our results reveal that treatment with RES decreases both the plasma TSH concentration and the hypothalamic TRH mRNA expression in SCH rats without increasing the plasma concentration of fT4. Although the specific mechanism remains unknown, this effect might be partly attributable to the capacity of RES to regulate TSH secretion by manipulating the levels of SIRT1 (34).

The OFT provides simultaneous measures of locomotion, exploration, and anxiety (26). In this study, the untreated model rats showed a decrease in the total distance traveled and the frequency of rearing in the OFT, which was increased in the SCH rats treated with RES, indicating that RES treatment improved the decreased locomotor activity and exploration in the untreated model rats. Moreover, the decreased total distance traveled and frequency of rearing indicated a higher level of anxiety in the untreated model rats, which may also have been reduced by the treatment with RES and LT4. The SPT is commonly used to assess anhedonia, which is a prominent symptom of depression in rodents. Immobility in the FST is taken as an index of despair behavior, which is another prominent symptom of depression. In this study, the SCH rats showed decreased sucrose preference index in the SPT and increased immobility in the FST, indicating a depression-like behavior in SCH rats, which was consistent with the findings in our previous study (6). Moreover, Detke et al. found that antidepressant drugs, which inhibit norepinephrine reuptake (desipramine or reboxetine) effectively, reduced immobility and selectively increased climbing behavior without affecting swimming, whereas the selective serotonin reuptake inhibitor (SSRI), which works through the serotonin system, reduced immobility and selectively increased swimming, without affecting climbing. In this study, our results showed that both RES and LT4 reduced immobility behaviors in the model rats (35). Thus, it is possible that RES might have an analogous therapeutic effect with the SSRIs in certain types of depression. Further studies are in need to verify the reliability of this hypothesis.

Treatment of thyroid dysfunction could reduce the psychiatric symptoms of depression in general (36), and clinical studies have shown an improvement in depressive-like symptoms after treatment with LT4 in SCH patients (37, 38). Thus, LT4 was selected as a positive control in this study. The results showed the expected antidepressant effect of LT4 on the SCH rats. Similarly, RES-treated rats showed an increased sucrose preference index and decreased immobility in the FST. These results indicate that treatment with RES also alleviated the depression-like behavior of the SCH rats.

Hyperactivity of the HPA axis is one of the most potent factors that trigger depression episodes (39), and abnormalities in HPA axis function are also well documented in rats with hypothyroidism (6). Similarly, the SCH rats displayed elevated adrenal mass, plasma corticosterone, and hypothalamic CRH mRNA expression. However, these changes were ameliorated by treatment with RES. Although RES reportedly stimulated cortisol biosynthesis and secretion in H295R adrenocortical cells *in vitro* (40), our results and other reports indicate that RES reduced the serum corticosterone concentration in stressed rats (11, 14) and unstressed mice (41). This discrepancy about the effect of RES on the HPA axis may be partly ascribed to the different experimental techniques and methods in the different studies.

Studies on human brains have evaluated the levels or activity of total GSK-3β protein in the prefrontal cortex in mood disorders, including depression (22, 42), and increasing evidence suggests that inhibition of GSK-3β might contribute to antidepressant activity (43). Consistent with the report that the enzymatic activity of GSK-3β was increased in depressed suicide victims (42), lower pGSK-3β and pGSK-3β/GSK-3β levels in the hippocampus were found in the SCH rats, which could induce depression-like behavior in this study (6). However, the increased GSK-3β levels were improved by the treatment with RES. L803-mts, a known GSK-3β inhibitor (24), induced an anti-immobility effect in the FST. Moreover, a lower GSK-3β level expressed in heterozygotic GSK-3β^+/−^ mice was associated with reduced immobility time in the FST (44). These results suggest a role for GSK-3β in the antidepressive-like effects of RES and highlight GSK-3β as a potential target in the treatment of SCH-associated depression.

β-catenin, a substrate of GSK-3β (45), has been implicated in brain development, cognitive activity, and dendritic growth (46). Phosphorylation of β-catenin by GSK-3β enhances the degradation of the protein, whereas phosphorylation of GSK-3β stabilizes β-catenin and promotes its accumulation in the cell cytoplasm. The unphosphorylated β-catenin can then migrate into the nucleus, where it associates with transcription factors to stimulate gene expression (47). β-catenin protein levels were lower in the postmortem prefrontal cortices of depressed subjects compared to non-depressed controls (22), and β-catenin levels in the hippocampus can serve as a marker for antidepressant behavior (48). Consistent with these results, the protein levels of p-β-catenin and p-β-catenin/β-catenin were upregulated in the hippocampus of the SCH rats, indicating β-catenin degradation. Together with these reports, the findings from this study reinforce the clinical observation that depressed subjects display a high GSK-3β activation state and low β-catenin levels (22).

Recent studies have demonstrated that cyclin D1 and c-myc, critical genes involved in cell proliferation and differentiation, were important target genes of the Wnt signaling pathway. Overexpression of cyclin D1 and c-myc is highly associated with the accumulation of β-catenin and mutational defects of the Wnt signaling pathway (49). In this study, protein levels of cyclin D1 and c-myc were decreased due to the increased β-catenin protein levels in the SCH rats, which was improved by RES treatment, further confirming the therapeutic effect of RES. Several recent studies have focused on the relationship between β-catenin and cyclin D1 or c-myc. For instance, a strong correlation was reported between β-catenin deregulation and cyclin D1 expression in primary colorectal tumors (50). Brabletz et al. (51) also reported a tight correlation between nuclear β-catenin accumulation and c-myc expression in colorectal adenomas. In this study, the results of Pearson’s correlation test suggested a significant positive association between the expression of p-β-catenin/β-catenin and cyclin D1 or c-myc. Based on our findings, the abnormal expression of β-catenin and its associated dysfunction of cyclin D1 and c-myc may play a key role in SCH-associated depression.

In this study, we first observed that RES alleviates depression-like behavior in SCH rats, which may be due to the regulation of HPA and HPT axes and the activity of the Wnt/β-catenin pathway in the hippocampus. Consistent with reports that RES does not cause adverse effects (15, 52), our results confirm that RES does not cause hyperthyroxinemia, which is a potential therapeutic advantage over LT4 treatment. However, this study presented several limitations. First, RES was administered only in a single dose. Thus, its dose relationship and long-term side-effects should be determined using different doses in future studies. Second, considering our previous finding that RES could alleviate the depression-like behavior of CUMS rats, together with the reports that RES treatment does not cause adverse effects (15, 52), we did not administer RES to the sham rats, which might make it subtly difficult to interpret many of the results. Third, although LT4 treatment was selected as a positive control in this study, the effect of RES on the depression-like behavior of the SCH rats was not compared with that of conventional antidepressant medications, such as fluoxetine.

In conclusion, our results demonstrate that RES improved anxiety- and depression-like behavior in SCH rats. This effect may be due, at least in part, to regulation of the HPA and HPT axes and the Wnt/β-catenin pathway in the hippocampus. Compared with the possible adverse effects of LT4 treatment, including cardiovascular events and symptoms associated with excess thyroid hormone, such as nervousness and palpitations (53), the credible efficacy with high safety margins (54, 55) of RES make it a promising candidate for the treatment of SCH-associated depression.

## Author Contributions

Associate Prof. Jin-Fang Ge and Prof. Fei-Hu Chen designed the study, and wrote the protocol and the first draft of the manuscript. Prof. Jin-Fang Ge and Dr. Ya-Yun Xu managed the literature searches and the statistical analyses. Ya-Yun Xu, Gan Qin, and Jiang-Qun Cheng performed animal model experiments. All authors contributed to and have approved the final manuscript.

## Conflict of Interest Statement

The authors declare that the research was conducted in the absence of any commercial or financial relationships that could be construed as a potential conflict of interest.
